# Importance of Fluctuating Amino Acid Residues in Folding and Binding of Proteins

**Published:** 2019

**Authors:** Renganathan Senthil, Singaravelu Usha, Konda Mani Saravanan

**Affiliations:** 1. Department of Bioinformatics, Faculty of Biosciences, The Marudupandiyar Institutions, Thanjavur-613403, Tamil Nadu, India; 2. Department of Bioinformatics, Bharathiar University, Coimbatore-641046, Tamil Nadu, India

**Keywords:** Amino acid, Binding sites, Cell-penetrating peptides, DNA-binding proteins

## Abstract

**Background:**

Conformational flexibility of proteins remains as one of the major events in protein-protein/DNA/ligand/small molecule binding to achieve its biological function in the cell. The availability of high-resolution structures of protein complexes is a valuable resource for researchers to understand the mechanisms behind such interactions and it is found that the flexibility of amino acid residues at binding sites is crucial for many important functions in the cell.

**Methods:**

In this article, our statistical method (PreFRP) developed based on fluctuating amino acid residues and various amino acid indices related to flexibility/rigidity were used to study the importance of fluctuating amino acid residues in thermonucleases from pathogenic bacteria, cell penetrating peptides and intrinsically disordered proteins responsible for many neural disorders.

**Results:**

The results from our analysis reveal the importance of fluctuating amino acid residues in folding and binding of proteins. The role of moderate and high fluctuating residues in themonucleases, cell penetrating peptide and disordered regions are discussed in detail.

**Conclusion:**

Therefore, our analysis will help in understanding the importance of fluctuating amino acid residues in proteins which undergo a conformation change phenomenon.

## Introduction

Proteins are functional units of the cell and play many important roles in various cellular processes like transport, metabolism, and signaling. The collection of proteins within a cell determines its health and function and the proteins in the cell interact with other proteins/DNA/small molecules to perform biological functions. During these interactions, the amino acid residues at the interface/binding site alter conformation in such a way to attain stability 1. Recognizing or identifying the binding sites in a protein is one of the major goals of bioinformatics. It was believed that the extraction of biologically relevant features of the binding sites of proteins may ultimately lead to accurate prediction of binding sites 2. The problem with this kind of approach is due to lack of our understanding of what investigated homologous subsequences in the 3D structure of proteins and reported surprising structural adaptability of identical subsequences 3. Wilson *et al* stated that common sequences of up to eight residues do occur in unrelated proteins and sequence-specific antibodies can be generated to test binding to identical sequences contained in unrelated proteins 4. Argos has examined the most frequently observed residue substitutions and their correlation with structural changes in the oligopeptide pairs of identical pentapeptides in unrelated proteins which yielded a possible guide for site-directed mutagenesis experiments when no tertiary structural information is at hand 5.

Minor and Kim have designed an 11 amino acid sequence (chameleon sequence fragment) that folds as an alpha helix in one position and beta sheet at another position of the IgG binding domain of the protein and they demonstrated that non-local interactions can determine the secondary structure of peptide sequences of substantial length 6. After a careful study, Dalal and Regan demonstrated careful selection of key amino acid residues to manipulate the balance of short and long-range interactions which stabilize either a helical or sheet conformation 7. Using all the knowledge provided by the various protein science research groups, it is clear that the fluctuating amino acid residues at binding sites or at certain positions are important for folding and binding of proteins.

## Materials and Methods

### Definition of fluctuating residues and amino acid indices

The definition of high, moderate and weak fluctuating amino acid residues by Ruvinsky *et al* 8 is presented in table 1 and is used in this study. The five highly fluctuating amino acid residues are glycine, alanine, serine, proline and aspartic acid, respectively. Glycine and ala-nine are two small hydrophobic amino acids as the most flexible molecules in proteins. Serine contains an OH group in side chain which can form a hydrogen bond, whereas aspartic acid contains a carboxylic acid in side chain which can lose a proton to give a negatively charged COO^−^. The role of five fluctuating residues in protein structure, folding and stability has been revealed in our previous studies 9,10.

**Table 1.** Fluctuating amino acid residues: High, moderate and weak fluctuating amino acid residues

**Fluctuating type**	**Amino acid residues**
High fluctuating amino acid residues	Glycine, Alanine, Serine, Proline and Aspartic acid
Moderate fluctuating amino acid residues	Threonine, Glutamic acid, Asparagine, Lysine, Cysteine, Glutamine, Arginine and Valine
Weak fluctuating amino acid residues	Histidine, Leucine, Methionine, Isoleucine, Tyrosine, Phenylalanine and Tryptophan

Hydrophobicity as a physicochemical property is effectively used to characterize secondary structures of proteins and it is considered as a dominant force for protein folding 9 and hence, 70 amino acid indices from AA index database (https://www.genome.jp/aaindex/) were the focus of this study. The indices related to hydrophobicity which reflect flexibility and rigidity were carefully selected from database. The twenty amino acid residues are written in order (higher to lower values of their property) to understand the contribution of fluctuating amino acid residues in proteins. The position-specific matrix of each amino acid residue in the indices is given in table 2.

**Table 2.** Position profiles of amino acid residues in various hydrophobicity indices

**Position**	**A**	**C**	**D**	**E**	**F**	**G**	**H**	**I**	**K**	**L**	**M**	**N**	**P**	**Q**	**R**	**S**	**T**	**V**	**W**	**Y**
**1**	0	16	1	1	7	3	0	11	2	2	1	0	0	1	10	0	0	2	10	3
**2**	1	4	5	3	10	0	1	12	5	5	2	1	0	0	1	0	0	9	7	4
**3**	0	1	3	3	12	1	1	11	3	5	4	2	0	2	2	0	0	9	7	4
**4**	0	4	0	5	8	1	0	7	1	12	4	5	5	2	2	2	2	6	3	1
**5**	3	3	1	1	6	0	3	3	2	15	2	3	1	4	0	3	0	7	6	7
**6**	2	2	5	1	6	1	1	3	2	9	13	2	2	2	2	0	0	10	2	5
**7**	8	3	1	1	3	1	5	2	3	0	13	2	4	4	0	3	3	5	5	4
**8**	7	7	0	2	1	8	3	0	0	2	9	1	2	1	2	2	7	1	3	12
**9**	6	4	0	2	0	3	10	1	6	1	3	1	6	2	2	4	7	3	4	5
**10**	16	6	1	2	1	9	5	1	0	1	1	0	3	2	0	7	8	0	1	6
**11**	9	3	2	2	0	14	9	2	0	3	0	3	5	1	1	6	7	0	2	1
**12**	4	3	0	5	0	3	6	0	5	1	1	3	3	1	4	3	17	2	4	5
**13**	3	1	3	3	0	3	8	2	5	0	5	2	7	2	9	7	2	1	3	4
**14**	3	1	8	5	0	3	6	0	3	0	4	2	7	8	5	5	2	1	4	3
**15**	1	4	5	4	1	3	2	1	2	0	1	11	7	6	3	7	7	5	0	0
**16**	1	0	6	6	2	5	2	3	1	2	2	13	5	4	1	5	3	4	3	2
**17**	1	4	5	11	1	3	2	1	2	5	0	8	6	8	1	6	0	3	2	1
**18**	3	2	9	8	0	4	2	5	3	4	0	5	0	10	7	4	1	1	0	2
**19**	1	1	12	2	5	1	1	4	9	2	4	5	3	4	7	5	3	0	0	1
**20**	1	1	3	3	7	4	3	1	16	1	1	1	4	6	11	1	1	1	4	0

### Prediction and visualization of fluctuating residues

In our previous work, a web server “PreFRP” was developed to visualize and predict fluctuating amino acid residues in proteins 11. The web server gets a Protein Data Bank file or sequence file to perform prediction of fluctuating amino acid residues based on the carbon content in amino acid residues and classification of 20 amino acid residues into high (G, A, S, P, and D), moderate (T, E, N, K, C, Q, R, and V), and weak fluctuating residues (H, L, M, I, Y, F, and W). The program assigns the index to three different groups of fluctuating amino acid residues as follows: For high fluctuating residues, the index is −2 and for moderate fluctuating residues, the index is −1 and for weak fluctuating residues, the index is 2.

### Fluctuating residues in thermonuclease

For almost three decades, *Staphylococcus aureus (S. aureus)* has been used as a model system to understand various functions in the cell and hence, the importance of fluctuating amino acid residues in thermonucleases has been studied in this research. A dataset of 127 thermonuclease protein structures was retrieved from Protein Data Bank 12. A uniprot search was made by using 1A2T (thermonuclease in *S. aureus*) as a reference thermonuclease which resulted in 127 protein structures with less than 3Å resolution. Fluctuating residues at different positions like helices in the dataset were computed by using PreFRP web server 11.

### Fluctuating residues in cell penetrating peptides

Many reports on structure and function of small molecules are available in the literature, but studies on peptides showing transport activity are very limited. In the present work, cell penetrating peptide, especially crotamine structure was investigated by computing composition of fluctuating amino acid residues. The peptide sequence and structure were retrieved from Protein Data Bank (PDB ID: 1H5O). The length of the peptide was 42 amino acid residues made up of one alpha helix, two antiparallel sheets, and three disulfide bridges.

### Fluctuating residues in intrinsic disordered proteins

The proteins with varying length (86 entries) made up of long disordered regions (greater than 30 amino acid residues) were downloaded from disprot database 13. Sequence based analysis of intrinsic disordered regions in proteins has revealed that the fluctuating amino acid residues plays vital role in promoting disorder 14. The predictors such as Glob plot 15, Ronn 16 and Pondr 17 were employed to perform predictions to compare with PreFRP results.

## Results

### Fluctuating residues in amino acid indices

The amino acid indices rank twenty amino acid residues based on their important physicochemical properties which contribute to protein folding. In the present work, seventy of such indices which define rigidity or flexibility of amino acid residues in the proteins were used. The position-specific scoring matrix of seventy indices is shown in table 2. From table 2, it can be observed that the fluctuating amino acid residues like glycine, alanine, and serine occupy maximum space at the tenth and eleventh positions, whereas the aspartic acid lies maximally at the eighteenth and nineteenth position. Similarly, proline prefers to occupy the last ten positions. The location of fluctuating residues in position-specific scoring matrix clearly implies that the residues can form chameleon sequence regions which are responsible for many neural disorders 18.

### Fluctuating residues in thermonucleases

**Figure 1.**
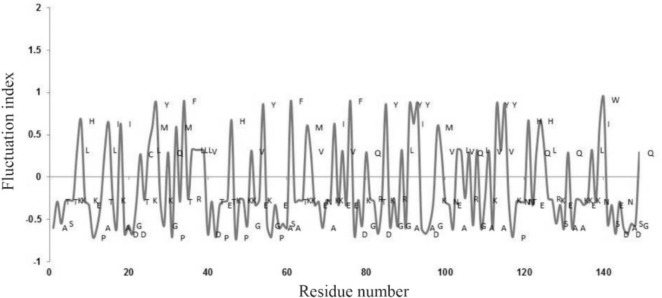
Fluctuating index for the amino acid residues along the sequences of thermonucleases.

The fluctuating index for the amino acid residues in thermonucleases along the sequence is shown in figure 1. The high and moderate fluctuating amino acid residues occur in higher percentages (28.9 and 49.1%). From our results, it is clear that the moderate fluctuating amino acid residues dominated in comparison to the other two types of fluctuating residues. Therefore, the distribution of high and moderate fluctuating residues helps the formation of secondary structure, mainly helices.

### Fluctuating residues in cell penetrating peptides

**Figure 2.**
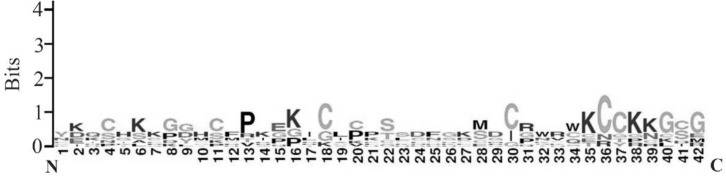
Amino acid composition in cell penetrating peptides.

**Figure 3.**
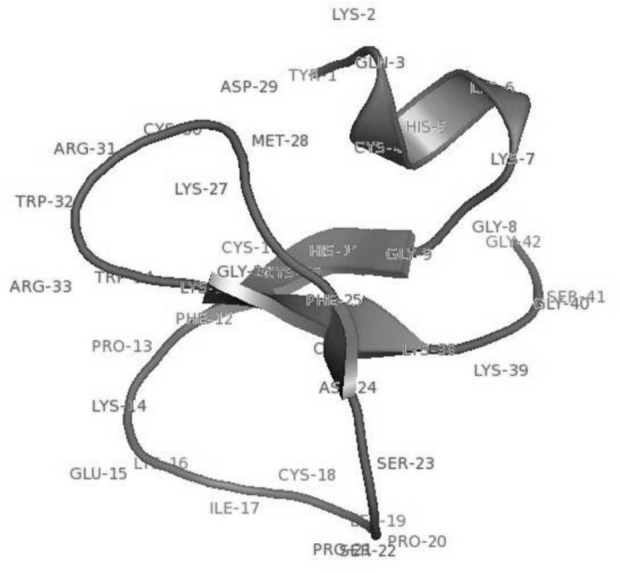
Random coil behavior of cell-penetrating peptides with more high fluctuating residues.

The amino acid composition of cell penetrating peptides is shown in figure 2. Comparison between amino acid compositions of cell penetrating peptides reveals the higher occurrences of high fluctuating amino acid residues. In figure 3, the lack of formation of secondary structure is evident due to the presence of high fluctuating amino acid residues in cell penetrating peptides. The random coil state of the peptides will form a regular secondary structure like helix or strands while binding with protein/DNA. Interestingly, the cell penetrating peptides are rich in cysteine, which can form disulfide bridges and contribute to the stability of peptides.

### Fluctuating amino acid residues in intrinsic disordered proteins

**Figure 4.**
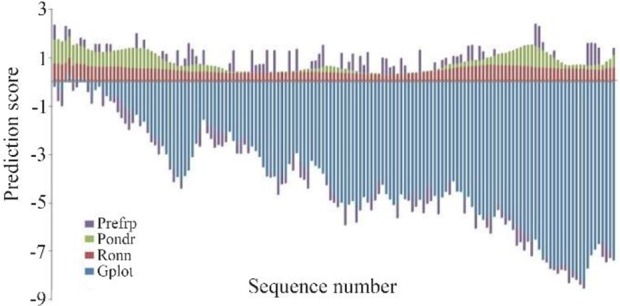
Comparison of prediction scores of four different predictors.

Analysis of sequence composition of ordered and disordered proteins has shown the difference and defines tertiary structures with fundamental knowledge for understanding molecular assembly and protein folding 14. The high fluctuating residues dominated in the case of sequences of intrinsically disordered proteins, whereas moderate fluctuating residues dominated in other cases. The prediction scores of four different methods such as G plot, Ronn, Pondr and PreFRP, respectively were computed and compared. Both positive and negative numbers in PreFRP helped to classify the ranking system better than entirely negative or positive ones (Figure 4). The methods used for comparison and its scoring scheme are shown in table 3 and the results imply that PreFRP performs comparatively better prediction like other methods.

**Table 3.** Sensitivity and specificity of PreFRP compared with the established algorithm

**Tool/server**	**(sequence/structure)**	**Proposed method**	**Scoring (Negative/positive)**	**Specificity**
**Glob plot**	Sequence	Hypothesis/Propensity	−	Order/globularity and disorder
**Ronn**	Sequence	Neural network	+	Native disordered region
**Pondr**	Sequence	Neural network	+	Natural disordered region
**PreFRP**	Sequence/Extraction based on structure	Probability/carbon Atom propensity	Both	Flexibility and fold/unfold

## Discussion

Despite the explosive growth in the number of high-resolution 3D protein structures, a key challenge for protein scientists is to understand how the sequence of amino acid residue code for a particular fold. Statistical analysis of sequence compositions of native folded protein structures revealed the role of certain amino acid residues in regular (helix or extended) and irregular regions (coil-like states). The conformation change phenomenon is mainly driven by high fluctuating residues occur at irregular regions and these residues play important role in folding and binding. Many computational methods were developed to identify fluctuating residues which mainly rely on the sequence or structural similarity. However, the existing methods cannot achieve high accuracy due to less conservation of fluctuation residues in homologous sequences and hence, a statistical method (PreFRP) to predict the amino acid residues in proteins as high, moderate and weak fluctuating was developed in this study. The PreFRP method focuses on the effective relationship between protein sequence, ability to undergo conformation change and functionality of protein structures.

## Conclusion

In the present study, the higher composition of high and moderate fluctuating amino acid residues in thermonucleases, cell penetrating peptides and intrinsically disordered protein regions was identified. Moreover, the location of high fluctuating amino acid residues in various classical amino acid indices were analyzed and discussed which are classified based on flexibility and rigidity property. Through the article, the importance of fluctuating amino acid residues in conformation change phenomenon was explored which helps protein scientists to understand the role of fluctuating residues in folding and binding of proteins.
